# Whole blood first resuscitation and association of blood product utilization based on mechanism of injury

**DOI:** 10.1007/s00068-026-03266-6

**Published:** 2026-07-21

**Authors:** Jamie Hadley, Jeffrey Conner, Christopher Thomas, Kaysey Llorente, Aaron Gilani, Brian White, Preston Miller III, Andrew Nunn, Gregory Stettler

**Affiliations:** 1 Department of Surgery, Division of Trauma and Acute Care Surgery, Atrium Health Wake Forest Baptist Hospital, Medical Center Boulevard, Winston-Salem, NC 27157 USA; 2 Department of Surgery, Division of Acute Care Surgery, Vanderbilt University School of Medicine, Nashville, TN USA; 3 Department of Surgery, Atrium Health Wake Forest Baptist Hospital, Winston-Salem, NC USA; 4 Department of Biostatistics and Data Science, Wake Forest School of Medicine, Winston- Salem, NC USA

**Keywords:** Whole blood, Hemorrhage, Blunt trauma, Penetrating trauma

## Abstract

**Introduction:**

Studies evaluating the effect of whole blood (WB) vs. component-based resuscitation accounting for mechanism of injury are lacking. We sought to evaluate the effects of a WB first resuscitation strategy regarding mechanism of injury.

**Methods:**

This is a retrospective cohort study of trauma patients who received any blood product during resuscitation from January 2016 to November 2021. The primary outcomes were total blood volume utilized within 24 h and 30-day mortality. Multivariable gamma regression with a log link was used for blood volume and logistic regression for mortality. A likelihood ratio test evaluated whether injury mechanism modified the treatment effect.

**Results:**

Between January 2016 and November 2021, 1,013 injured patients received blood products (785 WB-first). We did not detect statistically significant effect modification by injury mechanism for either outcome (interaction *p* = 0.44 for blood volume, *p* = 0.25 for mortality). In multivariable gamma regression, WB-first resuscitation was associated with a ratio of mean total blood volume of 0.71 (95% CI 0.55 to 0.90, *p* = 0.005) relative to components, representing approximately 29% lower utilization. WB-first was not associated with 30-day mortality (OR 0.90, 95% CI 0.48 to 1.71, *p* = 0.73).

**Conclusions:**

A WB-first resuscitation strategy is associated with lower blood volume utilized within the first 24 h, without statistically significant effect modification by injury mechanism (though clinically meaningful mechanism-specific differences cannot be excluded). The mortality estimate was non-significant across all analytic approaches but varied in direction (OR 0.66 to 1.48) and was sensitive to handling of approximately 30% of patients with incomplete laboratory data. Further studies are needed to determine which populations most benefit from WB-based resuscitation.

**Supplementary Information:**

The online version contains supplementary material available at 10.1007/s00068-026-03266-6.

## Introduction

Uncontrolled hemorrhage is a leading cause of death following severe injury [1–3]. As such, significant work has focused on minimizing the deleterious effects of trauma-induced coagulopathy (TIC) [4], while also developing and refining resuscitation strategies aimed at improving mortality and blood product utilization. Much of this work has focused on resuscitation strategies using blood component therapy [5–8]. However, driven by military experience [9–12], there has been a resurgence in the utilization of whole blood (WB) in civilian trauma centers and recent studies have evaluated the use of WB as the primary resuscitative product following injury. While results have varied, evidence suggests a survival and transfusion volume benefit when utilizing WB compared with component therapy [13–20].

Mechanism of injury influences coagulopathy following injury, and there are varying coagulopathic profiles in blunt vs. penetrating injury mechanisms. These differences lead to a direct association with mortality outcomes, both early and late during a patient’s hospital course. For example, penetrating injuries are more likely associated with release of tissue plasminogen activator (tPA) due to hemorrhagic shock leading to hyperfibrinolysis while blunt mechanisms are more likely to result in significant tissue injury and have a propensity for increased levels of plasminogen activator inhibitor 1 (PAI-1) release which is associated with increased incidence of fibrinolysis shutdown [21–26]. These differences in coagulopathic profile have been shown to have an impact on complications and risk of death at varying time points during a patient’s convalescence following injury [27, 28]. Based on these data, patients may have various resuscitative needs based on their mechanism of injury. Two recent studies have evaluated the specific effects of mechanism of injury in patients who received WB or component therapy resuscitation, both reporting differential mortality effects by mechanism [29, 30].

While penetrating and blunt mechanisms are associated with a different pathophysiology following injury, the existing evidence on mechanism-dependent effects of WB resuscitation is limited and the studies differ in treatment definitions, populations, and analytic approaches. Our aim is to evaluate a WB-first resuscitation strategy in severely injured patients in blunt vs. penetrating mechanism of injury. We hypothesize that WB utilization will not be associated with worse outcomes in either blunt or penetrating injury but may have a differential effect on resource utilization based on mechanism.

## Methods

Trauma patients who received any blood products as part of their resuscitation from January 2016–November 2021 at our ACS-verified Level 1 trauma center were included. Prior to 2018, our institutional resuscitation practice was component based using a ratio of 1:1:1 (plasma: red blood cells: platelets). Starting in 2018, our institutional massive transfusion resuscitation protocol was transitioned to a WB-first strategy, where patients > 16 years old received at least 2 units WB prior to transitioning to component therapy. All WB is leukocyte reduced low-titer (1:200) type “O.” Our lab uses a 26-day shelf life, but WB is converted to components prior to day 26 if it remains unused to prevent waste. The approximate volume of blood components at our institution is as follows: WB is 500 mL, packed red blood cells (PRBC) is 300 mL, plasma is 300 mL, an apheresis of platelets is 325 mL, and 5 units of cryoprecipitate is 90 mL. Patients who received WB first were compared with those who received traditional component therapy. Treatment group assignment was based on whether any WB was administered during the first 24 h: all 785 patients in the WB-first group received at least one unit (500 mL) of WB, with 375 (47.8%) receiving two or more units ( > = 1,000 mL). No patients in the component group received any WB. There were no crossovers between groups. Total blood volume utilized within the first 24 h was calculated as the sum of all blood products administered (WB, PRBC, plasma, platelets, and cryoprecipitate).

Clinical data were abstracted from the trauma registry, blood bank registry, and electronic medical record. Demographic data collected included age, sex, Glasgow coma scale (GCS), arrival systolic blood pressure (SBP), arrival heart rate (HR), mechanism of injury, presence of severe traumatic brain injury (TBI), and injury severity score (ISS). Complications recorded included myocardial infarction, stroke, venous thromboembolism, acute respiratory distress syndrome, and acute kidney injury. Overall volume of blood components administered was recorded for each patient.

The primary outcomes were total blood volume utilized within the first 24 h and 30-day all-cause mortality. Differences in patient characteristics and outcomes between transfusion resuscitation protocols were evaluated through univariate and multivariable analyses. Continuous variables were summarized as median (IQR) and categorical variables as counts and percentages. The Wilcoxon rank-sum test was used for continuous variables and chi-squared test for categorical variables, with Fisher’s exact test used when expected cell counts were small.

Multivariable analyses included gamma regression with a log link for total blood volume utilized and logistic regression for 30-day mortality. The primary intervention was transfusion resuscitation protocol (WB-first vs. component). To test whether injury mechanism modified the effect of transfusion protocol, we compared models with and without a protocol x mechanism interaction term using a likelihood ratio test. If the interaction was significant then mechanism-specific transfusion resuscitation protocol effects were estimated using marginal means. If not, the reduced model without interaction was used to estimate the overall treatment effect for transfusion resuscitation protocol. Covariates were selected based on clinical relevance and established associations with trauma outcomes. All models adjusted for age, sex, international normalized ratio (INR), injury severity score (ISS), lactate, systolic blood pressure (SBP), heart rate, and hematocrit. The primary analysis excluded patients with severe traumatic brain injury (TBI), defined as AIS head score > = 3, following Cotton et al. [19]. A sensitivity analysis including patients with severe TBI was performed to assess robustness of findings.

Model fit was assessed using deviance residual plots, variance inflation factors (VIF), and restricted cubic spline tests for nonlinearity (Supplementary Appendix).

Supplementary analyses assessed robustness to missing data, confounding, and analytic assumptions. These included multiple imputation using chained equations (MICE) with delta-adjustment for missing-not-at-random departures, inverse probability of treatment weighting (IPTW) and propensity-score overlap weighting (ATO) to address confounding by era, unadjusted vs. adjusted comparisons, restriction to 24-hour survivors, mechanism-stratified estimates from the interaction model, a WB-dose sensitivity analysis stratifying the WB-first exposure into 1-unit and > = 2-unit subgroups, and a post-hoc power analysis. Full details are provided in the Supplementary Appendix. All tests were two-tailed with significance set at *p* < 0.05. R version 4.5.2 (R Foundation for Statistical Computing, Vienna, Austria) was used for all analyses. The study was approved by the Wake Forest University School of Medicine institutional review board (IRB) #00058468 and need for consent for participation in the study was waived by the IRB in accordance with the Declaration of Helsinki.

## Results

**Fig. 1:**
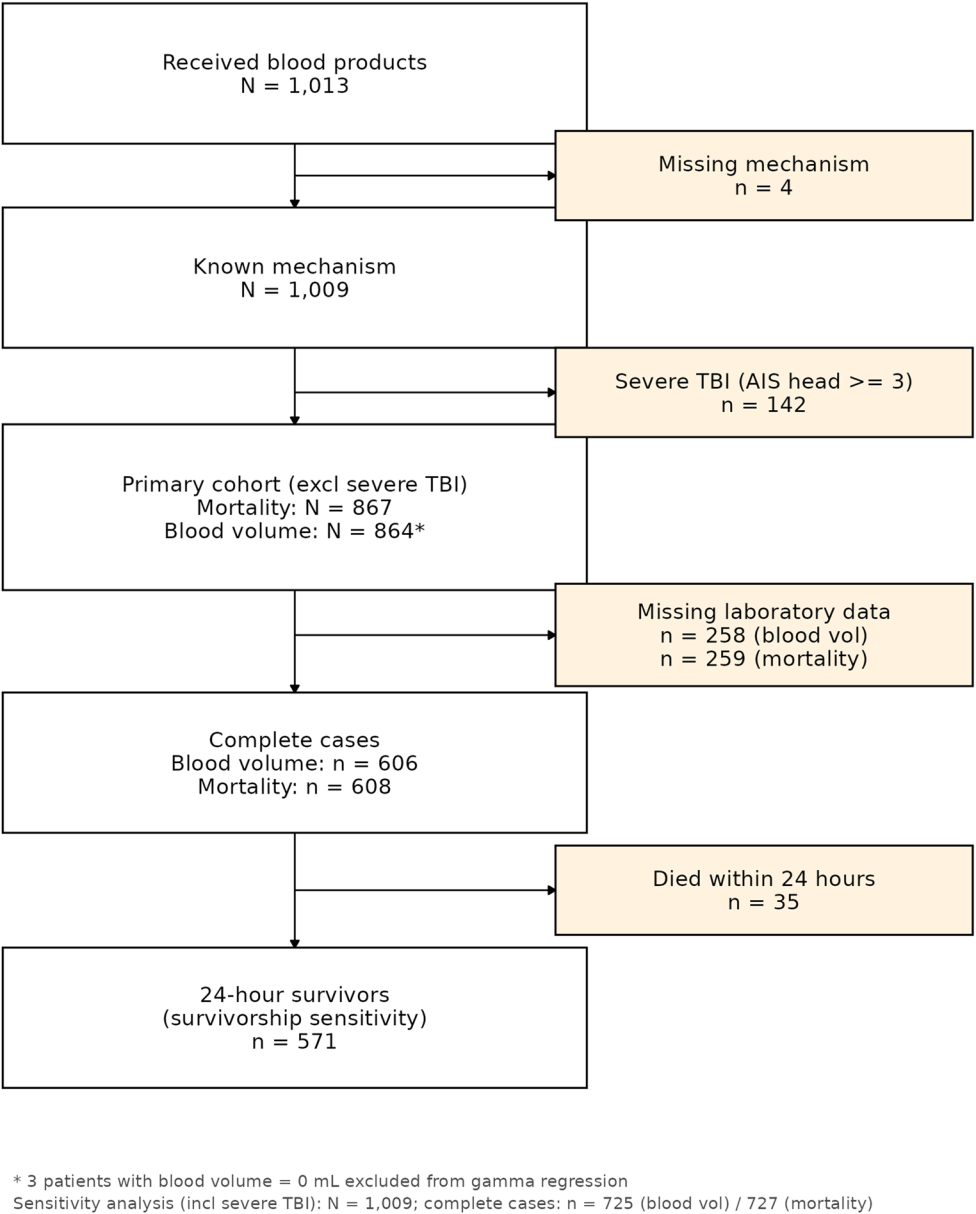
Cohort flow diagram. Of 1,013 patients who received blood products, 4 were excluded for missing mechanism data and 142 for severe TBI (AIS head > = 3), yielding a primary cohort of 867. Complete case analyses included 606 (blood volume) and 608 (mortality) patients after excluding those with missing laboratory data

Between January 2016 and November 2021, 1,013 patients received at least one unit of blood products. Of these, 785 (77%) received a WB-first resuscitation strategy and 228 (23%) received component therapy only. Four patients were missing mechanism of injury data, leaving 1,009 for mechanism-stratified analyses. Of the 1,009 patients with known mechanism, 142 had severe TBI (AIS head > = 3), yielding a primary analysis cohort of 867 (excluding severe TBI) and a sensitivity cohort of 1,009 (all patients). Three patients had zero total blood volume recorded and were excluded from the gamma regression, giving 864 eligible for the primary blood volume analysis. After exclusion of patients with missing laboratory data (hematocrit, lactate, INR, heart rate, or SBP), 606 complete cases were available for the primary blood volume model and 608 for the primary mortality model. Of the 606 blood volume complete cases, 571 survived past 24 h and comprised the survivorship bias sensitivity analysis (Fig. 1). Patient demographics and outcomes are summarized in Table 1. The WB-first group was older (median 50 vs. 41 years, *p* < 0.001), more likely to have a blunt mechanism of injury (75% vs. 61%, *p* < 0.001) and had a lower median ISS (22 vs. 27, *p* < 0.001). Arrival vitals (SBP, heart rate) and GCS were similar between groups. INR was lower in the WB-first group (1.09 vs. 1.13, *p* = 0.002). The WB-first group had shorter hospital length of stay (6 vs. 9 days, *p* < 0.001) and ICU length of stay (2 vs. 3 days, *p* = 0.003). Unadjusted 30-day mortality was 31% in the WB-first group and 24% in the components first group (*p* = 0.044). Median total blood products transfused were lower in the WB-first group (1,100 vs. 2,616 mL, *p* < 0.001).

Mechanism-stratified patient characteristics are presented in Supplementary Tables 1 and 2. Among blunt trauma patients (*n* = 726), the WB first group was older and had a lower ISS; median total blood products were lower in the WB first group (1,000 vs. 2,809 mL, *p* < 0.001). Among penetrating trauma patients (*n* = 283), differences in blood product utilization were not significant (1,589 vs. 2,319 mL, *p* = 0.11).

### Total blood volume utilized within the first 24 h

A gamma regression with log link was used to model total blood volume utilized. A likelihood ratio test for a protocol x mechanism interaction did not detect statistically significant effect modification (*p* = 0.44); main effects models are presented. In the primary analysis (excluding patients with severe TBI), WB-first resuscitation was associated with a ratio of mean total blood volume of 0.71 (95% CI 0.55 to 0.90, *p* = 0.005) relative to components only, and blunt mechanism was associated with a ratio of 0.70 (95% CI 0.54 to 0.89, *p* = 0.004) relative to penetrating. INR (1.85, 95% CI 1.32 to 2.70, *p* < 0.001), ISS (1.02, 95% CI 1.02 to 1.03, *p* < 0.001), lactate (1.07, 95% CI 1.03 to 1.11, *p* < 0.001), and hematocrit (0.98, 95% CI 0.97 to 1.00, *p* = 0.014) were also significant. Age, sex, SBP, and heart rate were not significant. In the sensitivity analysis (including all patients), results were similar: WB-first ratio 0.67 (95% CI 0.53 to 0.83, *p* < 0.001), blunt mechanism ratio 0.74 (95% CI 0.59 to 0.93, *p* = 0.010). Mechanism-stratified estimates from the interaction model showed a WB-first ratio of 0.66 (95% CI 0.49 to 0.89, *p* = 0.007) in blunt trauma and 0.80 (95% CI 0.55 to 1.18, *p* = 0.26) in penetrating trauma; the wider confidence interval in the penetrating subgroup reflects its smaller sample size. Full model results are presented in Table 2.

### 30-day all-cause mortality

Logistic regression was used to model 30-day all-cause mortality. A likelihood ratio test for the protocol x mechanism interaction did not detect statistically significant effect modification (*p* = 0.25); main effects models are presented. In the primary analysis (excluding patients with severe TBI), WB-first resuscitation was not associated with 30-day mortality (OR 0.90, 95% CI 0.48 to 1.71, *p* = 0.73). Injury mechanism was also not significant (blunt vs. penetrating: OR 0.76, 95% CI 0.37 to 1.57, *p* = 0.44). Age (OR 1.05, 95% CI 1.03 to 1.07, *p* < 0.001), lactate (OR 1.13, 95% CI 1.06 to 1.22, *p* < 0.001), ISS (OR 1.05, 95% CI 1.03 to 1.07, *p* < 0.001), and SBP (OR 0.99, 95% CI 0.98 to 1.00, *p* = 0.014) were significant predictors. Sex, INR, heart rate, and hematocrit were not significant. Mechanism-stratified estimates from the interaction model yielded a WB-first OR of 0.71 (95% CI 0.34 to 1.48, *p* = 0.36) in blunt trauma and 1.56 (95% CI 0.49 to 4.92, *p* = 0.45) in penetrating trauma. Neither stratum-specific estimate was significant, and the penetrating subgroup (*n* = 167, 19 events) was too small for reliable estimation. In the sensitivity analysis (including all patients), WB first remained non-significant (OR 1.14, 95% CI 0.69 to 1.92, *p* = 0.61). Full model results are presented in Table 3.

### Supplementary analyses

**Fig. 2:**
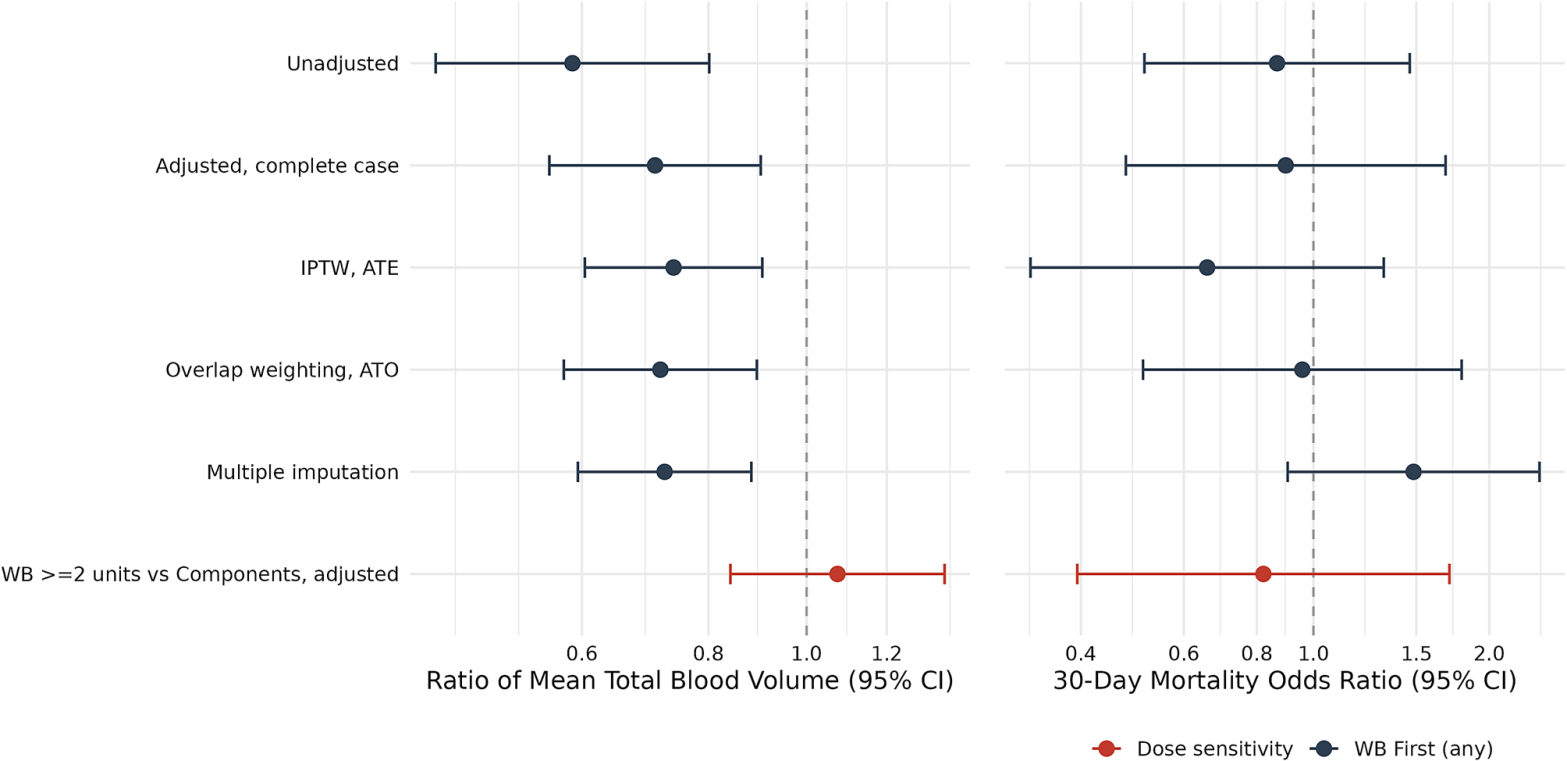
Treatment effect sensitivity analysis: WB first vs. component across analytic methods (primary analysis, excluding severe TBI). A dose-restricted row (WB > = 2 units vs. components first) is shown in red

Supplementary analyses including multiple imputation, MNAR delta-adjustment, IPTW, propensity-score overlap weighting (ATO), WB-dose stratification, and restriction to 24-hour survivors are detailed in the Supplementary Appendix with full results in Supplementary Tables 3–11. For blood volume, the WB-first treatment effect was consistent across all analytic approaches (ratios 0.59–0.74, all *p* < 0.01; Fig. 2). For mortality, the estimate was uniformly non-significant but varied widely in magnitude and direction (ORs 0.66–1.48), reflecting instability due to ~ 30% of patients excluded by complete case analysis and insufficient power (80% power to detect only OR < = 0.46 or > = 2.18). Model diagnostics were satisfactory: no multicollinearity (all VIF < 1.4), no systematic residual patterns, and no covariate nonlinearity after Bonferroni correction. Overlap weighting yielded a blood volume ratio of 0.72 (95% CI 0.58 to 0.89, *p* = 0.003) and a mortality OR of 0.96 (95% CI 0.51 to 1.79, *p* = 0.89), consistent with the complete-case estimates. In a sensitivity analysis stratifying the WB-first exposure into 1-unit and > = 2-unit subgroups, the blood-volume effect was concentrated in the 1-unit subgroup (ratio 0.43, 95% CI 0.34 to 0.55, *p* < 0.001), with no detectable difference for the WB > = 2-unit subgroup compared with components first (ratio 1.05, 95% CI 0.82 to 1.35, *p* = 0.67). Mortality was non-significant in both dose strata. The 1-unit subgroup had markedly lower baseline severity than other strata (median ISS 17 vs. 25, median lactate 2.8 vs. 3.6 mmol/L), and dose received is a post-treatment variable confounded by severity; this analysis characterizes treatment-effect heterogeneity rather than a causal dose-response.

## Discussion

After adjusting for clinical covariates, a WB-first resuscitation strategy was associated with approximately 29% lower total blood volume utilized within the first 24 h relative to component therapy. However, there was no effect of WB utilization found on mortality in blunt trauma patients or penetrating trauma patients.

Blunt and penetrating traumas create unique challenges following injury. The differing amount of local hemorrhage, tissue injury, and risk of traumatic brain injury can create variable pathophysiologic responses based on these mechanisms [22, 24–26]. The release of tPA, most closely associated with hemorrhagic shock and penetrating injuries, is the primary driver of hyperfibrinolysis [21, 23, 26]. On the other hand, a blunt mechanism of injury is more likely to result in significant tissue injury and have a propensity for increased levels of plasminogen activator inhibitor 1 (PAI-1) release, which is associated with increased incidence of fibrinolysis shutdown [24, 25]. This tissue injury also leads to increased rates of platelet activation, increasing the risk for thromboembolic complications following injury [27]. To underscore the importance of mechanism of injury to development of coagulopathy, a proposed clinical assessment tool developed by the Trans-Agency Consortium for Trauma-Induced Coagulopathy (TACTIC), denoted mechanism of injury as a modifier to their clinical coagulopathy score, in part due to the known differences in the coagulopathies seen in penetrating vs. blunt traumatic mechanisms [28].

Previous studies have evaluated WB resuscitation effects by mechanism of injury, both reporting differential mortality effects that we did not observe. In our study, the mortality estimate was non-significant across all analytic approaches but ranged from OR 0.66 (IPTW) to OR 1.48 (multiple imputation), with the direction reversing between complete-case and multiple-imputation analyses. This instability, together with the limited power for moderate mortality differences (post-hoc minimum detectable OR 0.46 or 2.18), means the mortality findings should be treated as inconclusive. Acharya et al. compared exclusive WB vs. exclusive component strategies in MTP-activated patients and found a significant interaction, with lower mortality in penetrating trauma patients receiving WB (RR 0.36, 95% CI 0.15 to 0.89) [29]. Dilday et al., in a prospective multicenter study, reported that LTOWB was associated with decreased mortality in penetrating trauma (OR 0.31, *p* < 0.05) but not in blunt trauma [30]. Several methodological differences may account for these discrepant findings. First, the treatment contrast differs both prior studies compared exclusive strategies, while we compared WB-first vs. component only protocols in which most WB-first patients also received subsequent component therapy, potentially attenuating any mechanism-dependent effect specific to exclusive WB. Second, the study populations differ in severity; Acharya et al. enrolled MTP-activated patients, while our broader inclusion captured a wider severity spectrum that would dilute effects concentrated among the most severely injured. Finally, Dilday et al. present stratified analyses but do not report a formal interaction test; observing significance in one subgroup but not another does not itself constitute evidence of effect modification.

Civilian trauma center applications for WB-first resuscitation have become increasingly more common [9–12]. Studies evaluating whole blood resuscitation strategies at several civilian trauma centers have resulted in encouraging outcomes, especially regarding reduced transfusion requirements [13–17, 20]. Our study supports previous findings and identifies the potential benefit associated with improved resource utilization in the form of lower transfusion volumes in those who receive WB [29]. The blood volume finding was robust across all supplementary analytic approaches, including multiple imputation, IPTW, and restriction to 24-hour survivors (all ratios 0.59–0.74, all *p* < 0.01; Fig. 2 and Supplementary Appendix).

Of the WB-first patients in the primary cohort, 58% received a single 500 mL unit of WB and 42% received > = 2 units. A sensitivity analysis stratified by dose received showed that the lower total blood volume associated with the WB-first protocol was concentrated in the 1-unit subgroup, which had markedly lower baseline severity (median ISS 17 vs. 25, lactate 2.8 vs. 3.6 mmol/L) than the components only comparator. Patients with injuries severe enough to require the protocol-as-intended dose of > = 2 units of WB did not use less total blood volume than components-first patients (ratio 1.05, 95% CI 0.82 to 1.35). Because dose received is a post-treatment variable confounded by severity, this analysis cannot disentangle a per-patient causal dose-response from heterogeneity of treatment effect; it does suggest that the population-level reduction in total transfusion volume under a WB-first protocol is consistent with earlier hemorrhage control in patients whose bleeding stops rapidly rather than with a uniform per-patient reduction across severity.

Our study has several limitations. This is a retrospective study with a quasi-experimental before-and-after design, and treatment assignment is nearly collinear with calendar time. We addressed potential time-related confounding with IPTW and propensity-score overlap weighting, which yielded consistent results for blood volume, but we cannot fully exclude residual confounding by era from unmeasured practice changes coincident with the protocol transition. Statistical adjustment was limited to observed covariates. Approximately 30% of eligible patients lacked laboratory values, and missingness was associated with higher mortality, suggesting a missing-not-at-random mechanism. For total blood volume, sensitivity analyses including MI with MNAR delta-adjustment did not change conclusions (ratios 0.69 to 0.73 across delta values, all *p* < 0.01). For 30-day mortality, the MI estimate reversed direction relative to the complete-case result (OR 1.48 under MAR; OR 1.29 to 1.49 across MNAR delta-adjustment from 0 to 1.5 SD, all non-significant); the mortality result is therefore highly sensitive to assumptions about the missing-data mechanism. MI cannot evaluate all possible missing data mechanisms (Supplementary Appendix). Changes in blood bank collection software may have led to incomplete capture of patients in the earlier study period.

In conclusion, while WB-first resuscitation strategy is inconclusive regarding survival, it was associated with lower total blood volume utilized within the first 24 h, with an estimated 29% lower utilization compared with component therapy. This difference was consistent across analytic approaches. Larger prospective studies will be needed to determine whether WB resuscitation is associated with a mortality benefit and whether this varies by mechanism of injury.

**Table 1:** Characteristics and outcomes of trauma patients who received any blood products as part of their resuscitation and comparison of patients who underwent a WB-first resuscitation strategy vs. a component strategy

	All patients (*N* = 1,013)	WB First (*N* = 785)	Components First (*N* = 228)	*p*-value
**Demographics**				
Age (years)	48 (31–65)	50 (32–67)	41 (27–57)	< 0.001
Male, n (%)	785 (77)	619 (79)	166 (73)	0.054
Blunt Mechanism, n (%)	726 (72)	587 (75)	139 (61)	< 0.001
Unknown	4	4	0	
SBP (mmHg)	100 (82–120)	100 (80–120)	102 (84–125)	0.19
Heart Rate (bpm)	95 (76–117)	94 (76–116)	101 (76–120)	0.15
Unknown	1	1	0	
GCS	14.0 (3.0–15.0)	14.0 (3.0–15.0)	15.0 (3.0–15.0)	0.49
Hematocrit (%)	37 (32–41)	37 (33–41)	36 (31–40)	0.016
Unknown	197	166	31	
Lactate (mmol/L)	3.40 (2.30–5.20)	3.30 (2.20–5.20)	3.61 (2.50–5.10)	0.20
Unknown	195	152	43	
INR	1.10 (1.03–1.21)	1.09 (1.03–1.20)	1.13 (1.05–1.27)	0.002
Unknown	189	165	24	
ISS	25 (14–34)	22 (14–34)	27 (18–41)	< 0.001
**Outcomes**				
Ventilator Days	1.0 (0.0–3.0)	1.0 (0.0–3.0)	1.0 (0.0–4.0)	0.001
ICU LOS (days)	2.0 (1.0–6.0)	2.0 (0.0–6.0)	3.0 (1.0–7.0)	0.003
Hospital LOS (days)	7 (2–15)	6 (2–14)	9 (4–18)	< 0.001
VTE, n (%)	21 (2.1)	18 (2.3)	3 (1.3)	0.44
MI, n (%)	3 (0.3)	3 (0.4)	0 (0)	> 0.99
Stroke, n (%)	18 (1.8)	15 (1.9)	3 (1.3)	0.78
AKI, n (%)	99 (9.8)	88 (11)	11 (4.8)	0.004
ARDS, n (%)	33 (3.3)	23 (2.9)	10 (4.4)	0.28
24-hr Mortality, n (%)	164 (16)	139 (18)	25 (11)	0.015
30-day Mortality, n (%)	294 (29)	240 (31)	54 (24)	0.044
**Blood Products Within First 24 h**				
Whole blood (mL)	500 (500–1,000)	500 (500–1,000)	0 (0–0)	< 0.001
PRBC (mL)	570 (0–1,464)	300 (0–1,118)	1,200 (600–2,400)	< 0.001
Plasma (mL)	0 (0–1,271)	0 (0–901)	1,225 (302–2,135)	< 0.001
Platelets (mL)	0 (0–202)	0 (0–0)	190 (0–332)	< 0.001
Cryoprecipitate (mL)	0 (0–0)	0 (0–0)	0 (0–0)	0.28
Total blood products (mL)	1,300 (500–3,379)	1,100 (500–2,861)	2,616 (1,136–4,971)	< 0.001

Data presented as median (IQR) or n (%). SBP=systolic blood pressure, HR=heart rate, GCS=Glasgow coma scale, INR=international normalized ratio, ISS=injury severity score, ICU=intensive care unit, LOS=length of stay, AKI=acute kidney injury, ARDS=acute respiratory distress syndrome, MI=myocardial infarction, VTE=venous thromboembolism, WB=whole blood, PRBC=packed red blood cells

**Table 2:** Multivariable gamma regression for total blood volume utilized within the first 24 h: primary analysis (*n* = 606 complete cases, excluding severe TBI) and sensitivity analysis (*n* = 725, all patients)

	Primary Analysis		Sensitivity Analysis	*p*-value
	Ratio of Means (95% CI)	*p*-value	Ratio of Means (95% CI)	
**Transfusion protocol**				
Components first	—		—	
WB first	0.71 (0.55 to 0.90)	0.005	0.67 (0.53 to 0.83)	< 0.001
**Injury mechanism**				
Penetrating	—		—	
Blunt	0.70 (0.54 to 0.89)	0.004	0.74 (0.59 to 0.93)	0.010
Age (years)	1.00 (1.00 to 1.01)	0.96	1.00 (1.00 to 1.01)	0.48
Sex: Male (vs. Female)	1.11 (0.87 to 1.41)	0.38	1.24 (0.99 to 1.54)	0.058
INR	1.85 (1.32 to 2.70)	< 0.001	1.32 (1.06 to 1.71)	< 0.001
ISS	1.02 (1.02 to 1.03)	< 0.001	1.02 (1.01 to 1.03)	< 0.001
Lactate (mmol/L)	1.07 (1.03 to 1.11)	< 0.001	1.08 (1.04 to 1.11)	< 0.001
SBP (mmHg)	1.00 (0.99 to 1.00)	0.053	1.00 (1.00 to 1.00)	0.13
Heart Rate (bpm)	1.00 (1.00 to 1.01)	0.093	1.00 (1.00 to 1.01)	0.047
Hematocrit (%)	0.98 (0.97 to 1.00)	0.014	0.97 (0.96 to 0.99)	< 0.001

See Fig. 1 for cohort derivation. Protocol x mechanism interaction not significant; main effects models presented. INR=international normalized ratio, ISS=injury severity score, SBP=systolic blood pressure

**Table 3:** Multivariable logistic regression for 30-day all-cause mortality: primary analysis (*n* = 608 complete cases, excluding severe TBI) and sensitivity analysis (*n* = 727, all patients)

	Primary Analysis		Sensitivity Analysis	*p*-value
	OR (95% CI)	*p*-value	OR (95% CI)	
**Transfusion protocol**				
Components first	—		—	
WB first	0.90 (0.48 to 1.71)	0.73	1.14 (0.69 to 1.92)	0.61
**Injury mechanism**				
Penetrating	—		—	
Blunt	0.76 (0.37 to 1.57)	0.44	0.72 (0.42 to 1.28)	0.26
Age (years)	1.05 (1.03 to 1.07)	< 0.001	1.04 (1.03 to 1.05)	< 0.001
Sex: Male (vs. Female)	1.26 (0.68 to 2.41)	0.47	1.27 (0.77 to 2.13)	0.36
Lactate (mmol/L)	1.13 (1.06 to 1.22)	< 0.001	1.12 (1.06 to 1.19)	< 0.001
INR	1.70 (0.94 to 3.13)	0.079	1.34 (1.04 to 1.79)	0.025
ISS	1.05 (1.03 to 1.07)	< 0.001	1.06 (1.04 to 1.08)	< 0.001
SBP (mmHg)	0.99 (0.98 to 1.00)	0.014	0.99 (0.99 to 1.00)	0.037
Heart Rate (bpm)	1.00 (0.99 to 1.01)	0.79	1.00 (0.99 to 1.01)	0.98
Hematocrit (%)	0.97 (0.93 to 1.01)	0.10	0.97 (0.94 to 1.00)	0.027

See Fig. 1 for cohort derivation. Protocol x mechanism interaction not significant; main effects models presented. INR=international normalized ratio, ISS=injury severity score, SBP=systolic blood pressure

## Supplementary Information

Below is the link to the electronic supplementary material.

Supplementary Material 1

## Data Availability

No datasets were generated or analysed during the current study.
