# Supplementary material for: Whole blood first resuscitation and association of blood product utilization based on mechanism of injury
Source: Eur J Trauma Emerg Surg. 2026 Jul 21;52(1):227. doi: 10.1007/s00068-026-03266-6 (PMC13388416; doi:10.1007/s00068-026-03266-6)
Supplement: Supplementary file 1 — Supplementary Material 1 [file 68_2026_3266_MOESM1_ESM.docx]

# Supplementary Appendix

## Supplementary Methods

### Model Diagnostics

Model fit for gamma regression was assessed using deviance residual plots. Multicollinearity was assessed using variance inflation factors (VIF). Linearity of continuous covariates on the log scale was evaluated by comparing models with linear terms to models with restricted cubic splines (4 knots).

### Supplementary Analyses

To assess the robustness of results, several supplementary analyses were conducted. First, multiple imputation using chained equations (MICE; m=20 imputations, 20 iterations, predictive mean matching for continuous variables, logistic regression for binary variables) was performed to address missing laboratory data (hematocrit, lactate, INR) under a missing-at-random (MAR) assumption. Pooled estimates were obtained using Rubin’s rules. Because the MAR assumption may not hold (patients with missing labs had higher mortality, suggesting a missing-not-at-random mechanism), a delta-adjustment sensitivity analysis was performed: after MI under MAR, imputed laboratory values for patients who died were shifted toward more extreme values (lower hematocrit, higher lactate, higher INR) by 0.5, 1.0, and 1.5 standard deviations to assess how results change under MNAR departures. Second, inverse probability of treatment weighting (IPTW) was used to address potential confounding related to the before-and-after study design. Propensity scores were estimated via logistic regression including all covariates; average treatment effect (ATE) weights were applied, and covariate balance was assessed using standardized mean differences (threshold <0.10). Robust (sandwich) standard errors were used for IPTW-weighted regression models. Third, unadjusted estimates were computed alongside adjusted estimates to quantify the impact of covariate adjustment. Fourth, a post-hoc power analysis estimated the minimum detectable odds ratio for mortality given the observed sample size, event rate, and treatment group proportions. Fifth, because total blood volume is measured within the first 24 hours, a survivorship bias sensitivity analysis restricted the gamma regression to patients surviving past 24 hours. Sixth, mechanism-stratified treatment effects were estimated from the interaction model using marginal means to characterize the direction and precision of the treatment effect within each mechanism subgroup, regardless of interaction significance. As an additional propensity-score-based sensitivity, overlap weighting^1^ was performed using the same propensity-score model. Overlap weights are bounded in [0, 0.25] by construction and target the equipoise population, avoiding the extreme-weight concern with IPTW. Robust (HC0) standard errors were used for ATO-weighted regressions. A WB-dose sensitivity analysis was performed in which the WB-first exposure was disaggregated into 1-unit and >=2-unit subgroups (components-first reference), matching the institutional protocol's 2-unit minimum, and a WB-dominant analysis classified patients by whether whole blood constituted more than 50% of total transfused volume.

## Supplementary Results

### Missing Data Assessment and Multiple Imputation

Hematocrit, lactate, and INR had substantial missing data (19.4%, 19.2%, and 18.7%, respectively). The primary analyses used complete cases, retaining 608 of 867 eligible patients (70.1%) for the mortality model (Figure 1). Patients with incomplete laboratory data had higher 30-day mortality (50.6%) compared with complete cases (15.3%), suggesting that missingness was not random (likely missing-not-at-random, as sicker patients may have died before labs were obtained). Treatment group composition was similar between complete and incomplete cases (WB first: 78.5% vs 80.3%).

Multiple imputation using chained equations (MICE; m=20 imputations, predictive mean matching for continuous variables, logistic regression for binary variables) was performed as a sensitivity analysis. MI assumes that data are missing at random (MAR), meaning missingness depends on observed variables but not on the unobserved values themselves. Given the association between missingness and mortality described above, the MAR assumption may not hold; the missing data pattern is more consistent with a missing-not-at-random (MNAR) mechanism. MI results should therefore be interpreted as sensitivity analyses under the MAR assumption, with the caveat that MNAR departures could further shift these estimates.

For total blood volume, the MI estimate was consistent with the complete case analysis (ratio of means 0.72, 95% CI 0.59 to 0.88, p=0.001 vs complete case 0.71). For 30-day mortality, however, the MI estimate diverged from the complete case result (MI: OR 1.48, 95% CI 0.90 to 2.44, p=0.12 vs complete case: OR 0.90, 95% CI 0.48 to 1.71, p=0.73). Both estimates are non-significant, but the reversal in direction reflects the sensitivity of the mortality analysis to missing data handling and the influence of the ~30% of patients excluded by complete case analysis.

Full MI model results are presented in Supplementary Tables 5 and 6.

### MNAR Delta-Adjustment Sensitivity

To assess sensitivity to MNAR departures, a delta-adjustment analysis was performed. After imputation under MAR, imputed laboratory values for patients who died were shifted toward more extreme values (lower hematocrit, higher lactate, higher INR) by increments of 0.5, 1.0, and 1.5 standard deviations, reflecting the assumption that the sickest patients had worse labs than MAR imputation predicts. For mortality, the treatment effect remained non-significant across all delta values (OR ranging from 1.49 at delta=0 to 1.29 at delta=1.5 SD, all p>0.10). For blood volume, results were similar (ratio ranging from 0.73 to 0.69, all p<0.002). The MNAR delta-adjustment did not change the conclusions for either outcome.

Results are presented in Supplementary Table 7.

### Inverse Probability of Treatment Weighting (IPTW)

Given the quasi-experimental before-and-after design, we performed an IPTW analysis to address potential confounding by era. Propensity scores were estimated using logistic regression with all covariates. All standardized mean differences were <0.10 after weighting, indicating adequate balance. The effective sample size was 461 (treated) and 92 (control). The IPTW-weighted gamma regression for blood volume yielded a ratio of means of 0.74 (95% CI 0.60 to 0.90, p=0.003; robust sandwich standard errors), consistent with the primary analysis. The IPTW-weighted logistic regression for mortality yielded OR 0.66 (95% CI 0.33 to 1.32, p=0.24; robust SEs), which was non-significant and had wide confidence intervals, reflecting reduced effective sample size after weighting.

### Unadjusted and Adjusted Estimates

To assess the impact of covariate adjustment, unadjusted estimates were computed on the same complete-case sample. For blood volume, the unadjusted ratio of means for WB first was 0.59 (95% CI 0.43 to 0.80, p<0.001), which attenuated to 0.71 (95% CI 0.55 to 0.90, p=0.005) after adjustment, suggesting that confounders partially explain the crude treatment difference. For mortality, both the unadjusted OR (0.87, 95% CI 0.51 to 1.46, p=0.59) and adjusted OR (0.90, 95% CI 0.48 to 1.71, p=0.73) were non-significant and similar in magnitude.

Results are presented in Supplementary Table 9.

### Treatment Effect Sensitivity

Supplementary Figure 3 summarizes the WB first treatment effect for blood volume across all analytic approaches, and Supplementary Figure 4 shows the corresponding mortality results. For blood volume, the treatment effect was consistent across unadjusted, adjusted, IPTW, and MI analyses (all ratios 0.59–0.74, all p<0.01). For mortality, the estimate was uniformly non-significant across all approaches but varied widely in magnitude and direction (ORs from 0.66 to 1.48), indicating instability and insufficient statistical information to draw conclusions about a mortality effect. A comparison of estimates across methods is presented in Supplementary Table 8.

### Model Diagnostics

Deviance residual plots for the gamma regression showed no systematic patterns; the Q-Q plot indicated slight right-skewness consistent with the heavy-tailed nature of transfusion volume data. Variance inflation factors for all model covariates were below 1.4 (range 1.10 to 1.37), indicating no multicollinearity.

Results are presented in Supplementary Table 3.

### Linearity Assessment

Restricted cubic spline tests for nonlinearity were conducted for each continuous covariate within the full model. For the blood volume model, evidence of nonlinearity was detected for age (p=0.047), heart rate (p=0.029), INR (p=0.013), and hematocrit (p=0.010). For the mortality model, no covariate demonstrated statistically significant nonlinearity (all p>0.05), though lactate was borderline (p=0.066). As a sensitivity analysis, the blood volume gamma regression was refit using restricted cubic splines (4 knots) for age, heart rate, INR, and hematocrit. The WB first treatment effect was slightly attenuated (ratio 0.77, 95% CI 0.62 to 0.97, p=0.02) compared with the linear specification (ratio 0.71, p=0.005), but remained significant. The spline model had a lower AIC (10,472 vs 10,508), suggesting improved fit, though the primary conclusion was unchanged.

Results are presented in Supplementary Table 4.

### Power Analysis

With 608 complete cases in the primary mortality analysis (93 events, 9.3 events per variable), the study had 80% power to detect an OR of <=0.46 or >=2.18 for the treatment effect. The study was therefore underpowered to detect clinically meaningful but moderate mortality differences (e.g., OR 0.7 or 1.5). In the penetrating trauma subgroup (n=167, 19 events), no detectable effect size achieved 80% power within a plausible range of ORs. The non-significant interaction test between protocol and mechanism should be interpreted cautiously given these power limitations.

### Survivorship Bias Sensitivity

Because total blood volume is measured within the first 24 hours, patients who die within that window contribute truncated volumes, potentially introducing survivorship bias into the blood volume analysis. As a sensitivity analysis, the gamma regression was restricted to patients surviving past 24 hours (n=571, excluding 35 early deaths from the 606 complete cases; Figure 1). The WB first treatment effect was similar (ratio 0.70, 95% CI 0.54 to 0.89, p=0.004 vs 0.71 in the full analysis), indicating that early mortality does not bias the blood volume estimate.

Results are presented in Supplementary Table 10.

### Mechanism-Stratified Estimates

Mechanism-stratified treatment effects were estimated from the interaction model using marginal means. For blood volume, the WB first ratio was 0.66 (95% CI 0.49 to 0.89, p=0.007) in blunt trauma and 0.80 (95% CI 0.55 to 1.18, p=0.26) in penetrating trauma. For mortality, the WB first OR was 0.71 (95% CI 0.34 to 1.48, p=0.36) in blunt trauma and 1.56 (95% CI 0.49 to 4.92, p=0.45) in penetrating trauma. The interaction was not significant for either outcome (p=0.44 for blood volume, p=0.25 for mortality).

Results are presented in Supplementary Table 11.

### Overlap Weighting (ATO)

Propensity-score overlap weights (ATO) were estimated using the same logistic propensity-score model as the IPTW analysis. Maximum observed weights were 0.95 (components-first) and 0.62 (WB-first), with no extreme values; the effective sample size was 368 (124 components-first, 356 WB-first) on a crude n of 606. All standardized mean differences were below 0.013 after weighting. The ATO-weighted gamma regression for blood volume yielded a ratio of means of 0.72 (95% CI 0.58 to 0.89, p=0.003; robust HC0 SE), consistent with the complete-case estimate of 0.71 and the IPTW estimate of 0.74. The ATO-weighted logistic regression for mortality yielded OR 0.96 (95% CI 0.51 to 1.79, p=0.89).

### WB Dose-Stratified Sensitivity

The WB-first exposure was disaggregated into 'WB 1 unit' and 'WB >=2 units' strata (components-first reference). The >=2-unit cutoff matches the institutional protocol specifying at least two units of whole blood prior to component therapy. Of the 477 WB-first patients in the primary cohort, 278 (58%) received exactly 1 unit and 199 (42%) received >=2 units. Baseline severity differed substantially across dose strata: the 1-unit subgroup had median ISS 17 (IQR 10 to 27) and lactate 2.8 mmol/L (2.0 to 4.3), compared with ISS 25 (16 to 41) and lactate 3.6 (2.5 to 5.1) in components-first patients and ISS 22 (14 to 30) and lactate 3.5 (2.3 to 5.3) in the WB >=2-unit subgroup. Adjusted gamma regression yielded a blood-volume ratio of 0.43 (95% CI 0.34 to 0.55, p<0.001) for WB 1 unit and 1.05 (0.82 to 1.35, p=0.67) for WB >=2 units; adjusted 30-day mortality ORs were 0.97 (0.49 to 1.94) and 0.82 (0.41 to 1.66), both non-significant. A per-step linear trend test (Components=0 / WB 1=1 / WB >=2=2) gave a blood-volume ratio of 1.12 per step (p=0.08) and a mortality OR of 0.90 per step (p=0.55). A separate WB-dominant analysis (WB >50% of total volume) gave a blood-volume ratio of 0.29 (0.24 to 0.34, p<0.001) and a mortality OR of 0.85 (0.43 to 1.71, p=0.66); the volume contrast is partially tautological because total volume is bounded by approximately twice the WB volume when WB is dominant. Dose received is a post-treatment variable confounded by severity; the analysis characterizes treatment-effect heterogeneity, not a causal dose-response.

### Supplementary Table 1. Blunt Trauma: WB First vs Component Strategy (N=726)

Data presented as median (IQR) or n (%).

|  | All (N=726) | WB First (N=587) | Components First (N=139) | p-value |
| --- | --- | --- | --- | --- |
| **Demographics** |  |  |  |  |
| Age (years) | 54 (36–68) | 56 (37–70) | 47 (32–60) | <0.001 |
| Male, n (%) | 530 (73) | 436 (74) | 94 (68) | 0.11 |
| SBP (mmHg) | 100 (82–122) | 100 (82–120) | 100 (84–125) | 0.60 |
| Heart Rate (bpm) | 95 (76–118) | 94 (76–116) | 101 (77–120) | 0.10 |
| GCS | 14.0 (3.0–15.0) | 14.0 (3.0–15.0) | 15.0 (3.0–15.0) | 0.87 |
| Hematocrit (%) | 37 (32–41) | 37 (32–41) | 35 (31–39) | 0.017 |
| Unknown | 124 | 107 | 17 |  |
| Lactate (mmol/L) | 3.20 (2.10–4.80) | 3.20 (2.10–4.80) | 3.20 (2.20–5.10) | 0.74 |
| Unknown | 126 | 100 | 26 |  |
| INR | 1.10 (1.04–1.22) | 1.09 (1.03–1.21) | 1.15 (1.05–1.31) | 0.001 |
| Unknown | 116 | 103 | 13 |  |
| ISS | 25 (16–38) | 22 (14–34) | 33 (19–43) | <0.001 |
| **Outcomes** |  |  |  |  |
| Ventilator Days | 1 (0–4) | 1 (0–4) | 2 (0–5) | <0.001 |
| ICU LOS (days) | 3.0 (1.0–7.0) | 3.0 (1.0–6.0) | 3.0 (1.0–8.0) | 0.011 |
| Hospital LOS (days) | 8 (3–16) | 7 (3–15) | 10 (4–19) | 0.006 |
| VTE, n (%) | 12 (1.7) | 11 (1.9) | 1 (0.7) | 0.48 |
| MI, n (%) | 3 (0.4) | 3 (0.5) | 0 (0) | >0.99 |
| Stroke, n (%) | 15 (2.1) | 13 (2.2) | 2 (1.4) | 0.75 |
| AKI, n (%) | 81 (11) | 73 (12) | 8 (5.8) | 0.024 |
| ARDS, n (%) | 28 (3.9) | 20 (3.4) | 8 (5.8) | 0.20 |
| 24-hr Mortality, n (%) | 104 (14) | 88 (15) | 16 (12) | 0.29 |
| 30-day Mortality, n (%) | 211 (29) | 174 (30) | 37 (27) | 0.48 |
| **Blood Products Within First 24 Hours** |  |  |  |  |
| Whole blood (mL) | 500 (500–1,000) | 500 (500–1,000) | 0 (0–0) | <0.001 |
| PRBC (mL) | 300 (0–1,200) | 280 (0–900) | 1,470 (600–2,400) | <0.001 |
| Plasma (mL) | 0 (0–1,179) | 0 (0–662) | 1,235 (297–2,425) | <0.001 |
| Platelets (mL) | 0 (0–185) | 0 (0–0) | 187 (0–350) | <0.001 |
| Cryoprecipitate (mL) | 0 (0–0) | 0 (0–0) | 0 (0–0) | 0.15 |
| Total blood products (mL) | 1,245 (500–3,151) | 1,000 (500–2,615) | 2,809 (1,165–5,054) | <0.001 |

SBP=systolic blood pressure, HR=heart rate, GCS=Glasgow coma scale, INR=international normalized ratio, ISS=injury severity score, ICU=intensive care unit, LOS=length of stay, AKI=acute kidney injury, ARDS=acute respiratory distress syndrome, MI=myocardial infarction, VTE=venous thromboembolism.

### Supplementary Table 2. Penetrating Trauma: WB First vs Component Strategy (N=283)

Data presented as median (IQR) or n (%).

|  | All (N=283) | WB First (N=194) | Components First (N=89) | p-value |
| --- | --- | --- | --- | --- |
| **Demographics** |  |  |  |  |
| Age (years) | 33 (25–50) | 34 (25–48) | 32 (24–51) | 0.84 |
| Male, n (%) | 251 (89) | 179 (92) | 72 (81) | 0.005 |
| SBP (mmHg) | 100 (78–119) | 98 (70–118) | 102 (86–124) | 0.061 |
| Heart Rate (bpm) | 97 (75–117) | 95 (75–116) | 99 (74–117) | 0.73 |
| GCS | 15.0 (3.0–15.0) | 14.5 (3.0–15.0) | 15.0 (3.0–15.0) | 0.29 |
| Hematocrit (%) | 38 (34–42) | 38 (34–42) | 38 (32–40) | 0.14 |
| Unknown | 73 | 59 | 14 |  |
| Lactate (mmol/L) | 4.2 (2.7–6.5) | 4.1 (2.6–6.7) | 4.2 (3.2–6.0) | 0.80 |
| Unknown | 69 | 52 | 17 |  |
| INR | 1.08 (1.02–1.18) | 1.07 (1.02–1.16) | 1.12 (1.03–1.24) | 0.15 |
| Unknown | 73 | 62 | 11 |  |
| ISS | 22 (13–29) | 22 (13–29) | 25 (17–34) | 0.16 |
| **Outcomes** |  |  |  |  |
| Ventilator Days | 1.00 (0.00–2.00) | 0.00 (0.00–2.00) | 1.00 (0.00–2.00) | 0.35 |
| ICU LOS (days) | 1.0 (0.0–4.0) | 1.0 (0.0–3.0) | 2.0 (1.0–5.0) | 0.003 |
| Hospital LOS (days) | 5 (1–11) | 4 (1–9) | 9 (3–15) | <0.001 |
| VTE, n (%) | 9 (3.2) | 7 (3.6) | 2 (2.2) | 0.72 |
| Stroke, n (%) | 3 (1.1) | 2 (1.0) | 1 (1.1) | >0.99 |
| AKI, n (%) | 18 (6.4) | 15 (7.7) | 3 (3.4) | 0.16 |
| ARDS, n (%) | 5 (1.8) | 3 (1.5) | 2 (2.2) | 0.65 |
| 24-hr Mortality, n (%) | 60 (21) | 51 (26) | 9 (10) | 0.002 |
| 30-day Mortality, n (%) | 82 (29) | 65 (34) | 17 (19) | 0.013 |
| **Blood Products Within First 24 Hours** |  |  |  |  |
| Whole blood (mL) | 500 (0–1,000) | 1,000 (500–1,000) | 0 (0–0) | <0.001 |
| PRBC (mL) | 600 (0–1,800) | 300 (0–1,733) | 1,146 (600–2,012) | <0.001 |
| Plasma (mL) | 604 (0–1,820) | 260 (0–1,520) | 1,217 (313–1,898) | <0.001 |
| Platelets (mL) | 0 (0–297) | 0 (0–276) | 190 (0–300) | 0.004 |
| Cryoprecipitate (mL) | 0 (0–0) | 0 (0–0) | 0 (0–0) | 0.77 |
| Total blood products (mL) | 1,742 (780–4,197) | 1,589 (500–4,317) | 2,319 (1,106–4,157) | 0.11 |

SBP=systolic blood pressure, HR=heart rate, GCS=Glasgow coma scale, INR=international normalized ratio, ISS=injury severity score, ICU=intensive care unit, LOS=length of stay, AKI=acute kidney injury, ARDS=acute respiratory distress syndrome, VTE=venous thromboembolism.

### Supplementary Table 3. Variance Inflation Factors for Primary Gamma Regression Model

| Variable | VIF |
| --- | --- |
| WB first | 1.10 |
| Mechanism | 1.34 |
| Age | 1.37 |
| Male | 1.14 |
| INR | 1.13 |
| ISS | 1.12 |
| Lactate | 1.24 |
| SBP | 1.13 |
| Heart rate | 1.12 |
| Hematocrit | 1.18 |

All VIF values < 1.4, indicating no multicollinearity.

### Supplementary Table 4. Restricted Cubic Spline Tests for Nonlinearity

| Variable | Model | LR chi-sq | df | p-value |
| --- | --- | --- | --- | --- |
| Age | Blood Volume (Gamma) | 8.33 | 2 | 0.047 |
| SBP | Blood Volume (Gamma) | 0.25 | 2 | 0.91 |
| Heart rate | Blood Volume (Gamma) | 9.42 | 2 | 0.029 |
| Lactate | Blood Volume (Gamma) | 4.22 | 2 | 0.20 |
| INR | Blood Volume (Gamma) | 11.20 | 2 | 0.013 |
| ISS | Blood Volume (Gamma) | 5.49 | 2 | 0.14 |
| Hematocrit | Blood Volume (Gamma) | 12.08 | 2 | 0.010 |
| Age | Mortality (Logistic) | 0.75 | 2 | 0.69 |
| SBP | Mortality (Logistic) | 0.09 | 2 | 0.95 |
| Heart rate | Mortality (Logistic) | 1.04 | 2 | 0.60 |
| Lactate | Mortality (Logistic) | 5.44 | 2 | 0.066 |
| INR | Mortality (Logistic) | 4.46 | 2 | 0.11 |
| ISS | Mortality (Logistic) | 1.40 | 2 | 0.50 |
| Hematocrit | Mortality (Logistic) | 1.30 | 2 | 0.52 |

Nonlinearity tests compare models with linear terms to models with restricted cubic splines (4 knots). None are statistically significant after Bonferroni correction (threshold p<0.0036 for 14 tests).

### Supplementary Table 5. Multiple Imputation: Gamma Regression for Total Blood Volume (m=20 Imputations, Rubin’s Rules)

| Term | Ratio of Means | 95% CI | p-value |
| --- | --- | --- | --- |
| WB first | 0.72 | 0.59 to 0.88 | 0.001 |
| Blunt | 0.69 | 0.57 to 0.83 | <0.001 |
| Age | 1.00 | 1.00 to 1.00 | 0.95 |
| Male | 1.12 | 0.92 to 1.36 | 0.26 |
| INR | 1.62 | 1.23 to 2.14 | <0.001 |
| ISS | 1.03 | 1.02 to 1.03 | <0.001 |
| Lactate | 1.08 | 1.05 to 1.11 | <0.001 |
| SBP | 1.00 | 0.99 to 1.00 | 0.008 |
| Heart rate | 1.00 | 1.00 to 1.01 | 0.043 |
| Hematocrit | 0.98 | 0.96 to 0.99 | 0.001 |

### Supplementary Table 6. Multiple Imputation: Logistic Regression for 30-Day Mortality (m=20 Imputations, Rubin’s Rules)

| Term | OR | 95% CI | p-value |
| --- | --- | --- | --- |
| WB first | 1.48 | 0.90 to 2.44 | 0.12 |
| Blunt | 0.70 | 0.43 to 1.13 | 0.14 |
| Age | 1.04 | 1.03 to 1.05 | <0.001 |
| Male | 1.22 | 0.76 to 1.94 | 0.41 |
| Lactate | 1.15 | 1.06 to 1.23 | <0.001 |
| INR | 1.91 | 1.05 to 3.47 | 0.034 |
| ISS | 1.05 | 1.03 to 1.06 | <0.001 |
| SBP | 0.98 | 0.98 to 0.99 | <0.001 |
| Heart rate | 0.99 | 0.98 to 1.00 | 0.002 |
| Hematocrit | 0.97 | 0.94 to 1.01 | 0.12 |

### Supplementary Table 7. MNAR Delta-Adjustment Sensitivity Analysis

Imputed laboratory values for patients who died were shifted by delta standard deviations toward more extreme values (lower hematocrit, higher lactate, higher INR).

**Panel A: 30-Day Mortality (OR for WB First)**

| Delta (SD) | OR | 95% CI | p-value |
| --- | --- | --- | --- |
| 0 | 1.49 | 0.91 to 2.44 | 0.12 |
| 0.5 | 1.45 | 0.87 to 2.42 | 0.15 |
| 1.0 | 1.38 | 0.81 to 2.34 | 0.23 |
| 1.5 | 1.29 | 0.75 to 2.22 | 0.35 |

**Panel B: Total Blood Volume (Ratio of Means for WB First)**

| Delta (SD) | Ratio | 95% CI | p-value |
| --- | --- | --- | --- |
| 0 | 0.73 | 0.60 to 0.89 | 0.002 |
| 0.5 | 0.72 | 0.59 to 0.87 | <0.001 |
| 1.0 | 0.70 | 0.58 to 0.85 | <0.001 |
| 1.5 | 0.69 | 0.57 to 0.84 | <0.001 |

### Supplementary Table 8. Treatment Effect Comparison Across Analytic Methods

| Analysis | Blood Volume Ratio (95% CI) | Mortality OR (95% CI) |
| --- | --- | --- |
| Unadjusted | 0.59 (0.43 to 0.80) | 0.87 (0.51 to 1.46) |
| Adjusted (complete case) | 0.71 (0.56 to 0.90) | 0.90 (0.48 to 1.68) |
| IPTW-weighted | 0.74 (0.60 to 0.90) | 0.66 (0.33 to 1.32) |
| Multiple imputation | 0.72 (0.59 to 0.88) | 1.48 (0.90 to 2.44) |

### Supplementary Table 9. Unadjusted vs Adjusted Estimates (Complete Cases)

| Model | Estimate | 95% CI |
| --- | --- | --- |
| Blood Volume (Unadjusted) | 0.59 | 0.43 to 0.80 |
| Blood Volume (Adjusted) | 0.71 | 0.56 to 0.90 |
| Mortality (Unadjusted) | 0.87 | 0.51 to 1.46 |
| Mortality (Adjusted) | 0.90 | 0.48 to 1.68 |

### Supplementary Table 10. Survivorship Bias Sensitivity Analysis: Gamma Regression for Total Blood Volume

| Analysis | N | WB First Ratio | 95% CI | p-value |
| --- | --- | --- | --- | --- |
| Primary (all patients) | 606 | 0.71 | 0.56 to 0.90 | 0.005 |
| 24-hour survivors | 571 | 0.70 | 0.55 to 0.89 | 0.004 |

### Supplementary Table 11. Mechanism-Stratified Treatment Effects (From Interaction Model)

| Outcome | Mechanism | Estimate | 95% CI | p-value | Interaction p |
| --- | --- | --- | --- | --- | --- |
| Blood Volume | Blunt | 0.66 | 0.49 to 0.89 | 0.007 | 0.44 |
| Blood Volume | Penetrating | 0.80 | 0.55 to 1.18 | 0.26 | 0.44 |
| Mortality | Blunt | 0.71 | 0.34 to 1.48 | 0.36 | 0.25 |
| Mortality | Penetrating | 1.56 | 0.49 to 4.92 | 0.45 | 0.25 |

Blood volume estimates are ratios of means (WB first / Components first). Mortality estimates are odds ratios.

## Supplementary Figures

### Supplementary Figure 1


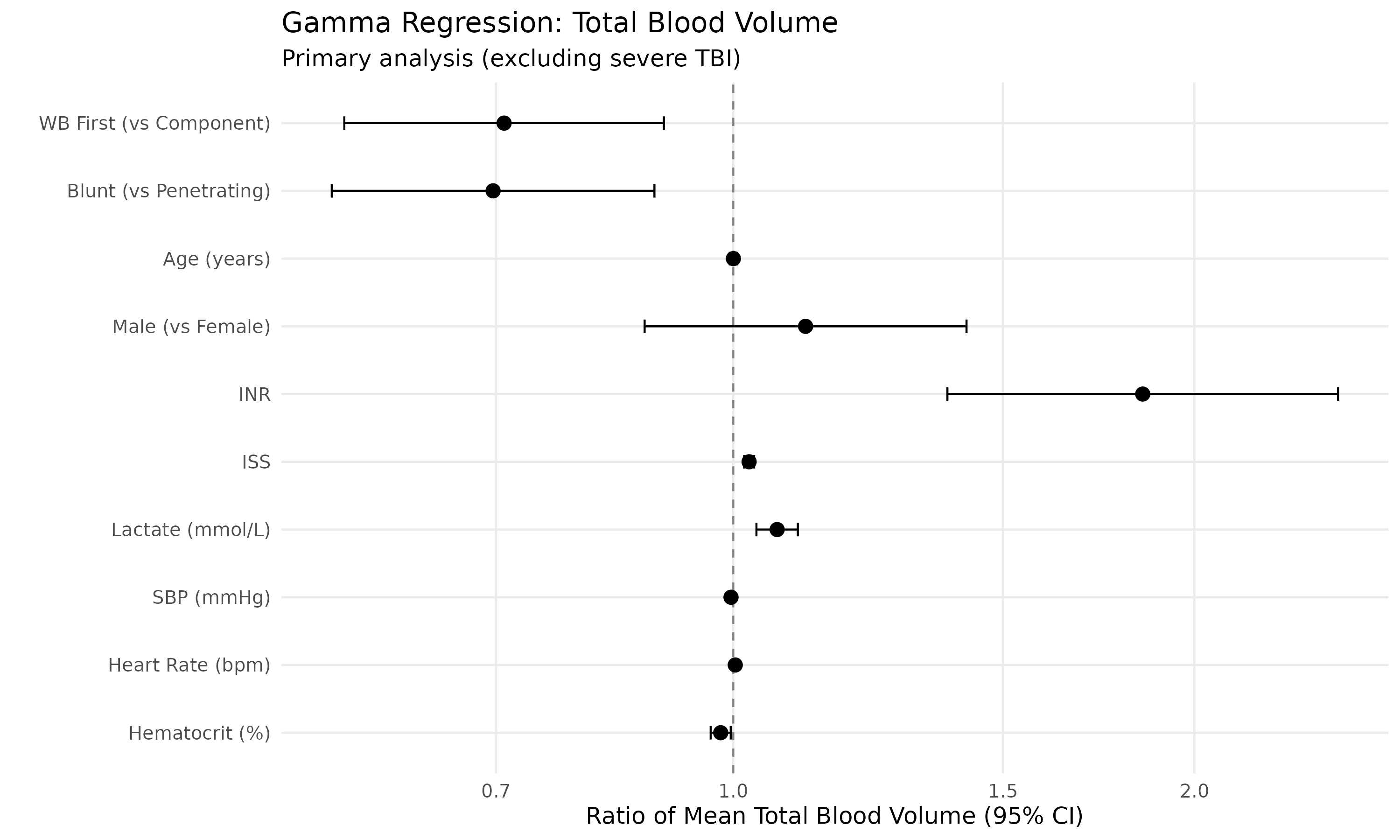


Supplementary Figure 1. Forest plot of gamma regression coefficients for total blood volume model (primary analysis, excluding severe TBI).

### Supplementary Figure 2


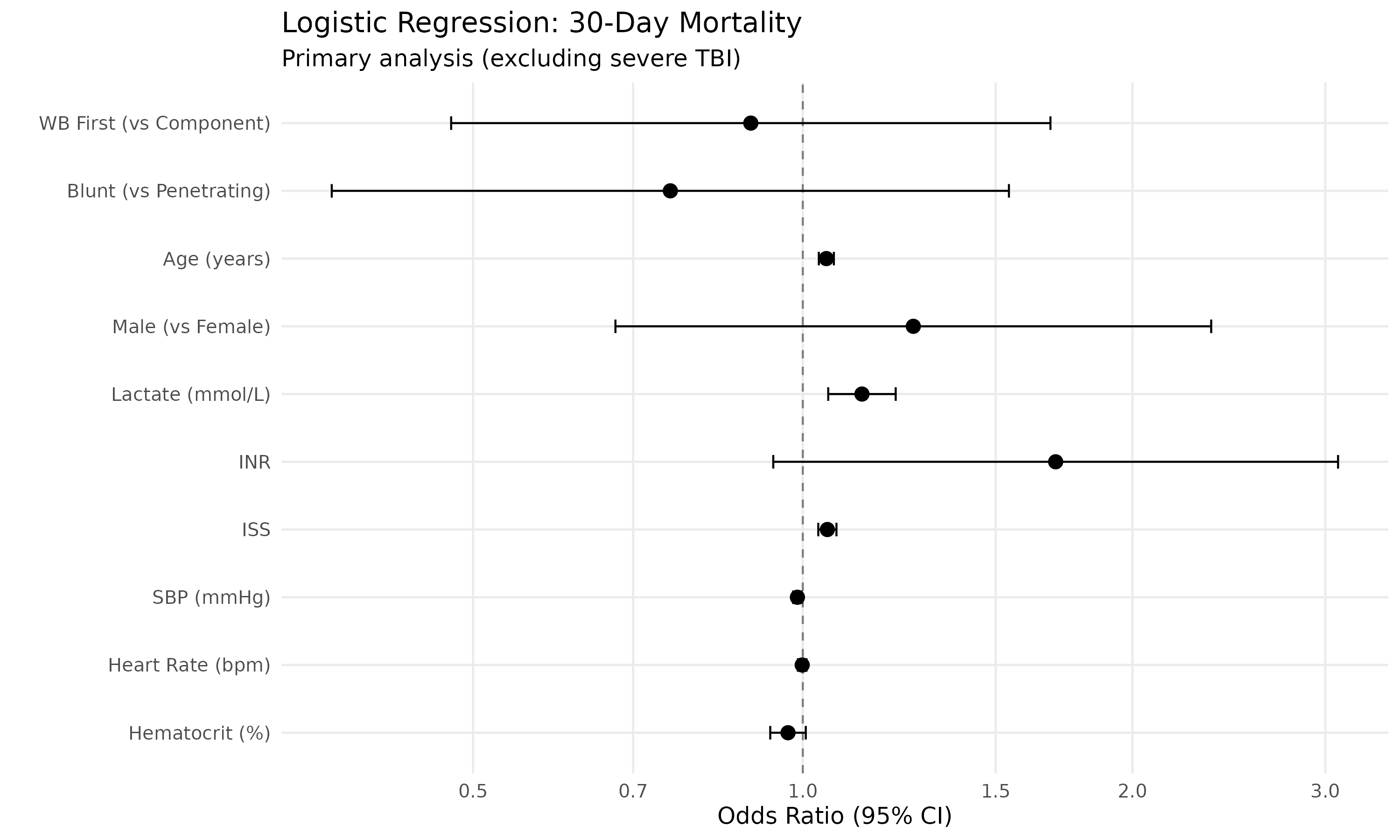


Supplementary Figure 2. Forest plot of logistic regression coefficients for 30-day mortality model (primary analysis, excluding severe TBI).

### Supplementary Figure 3


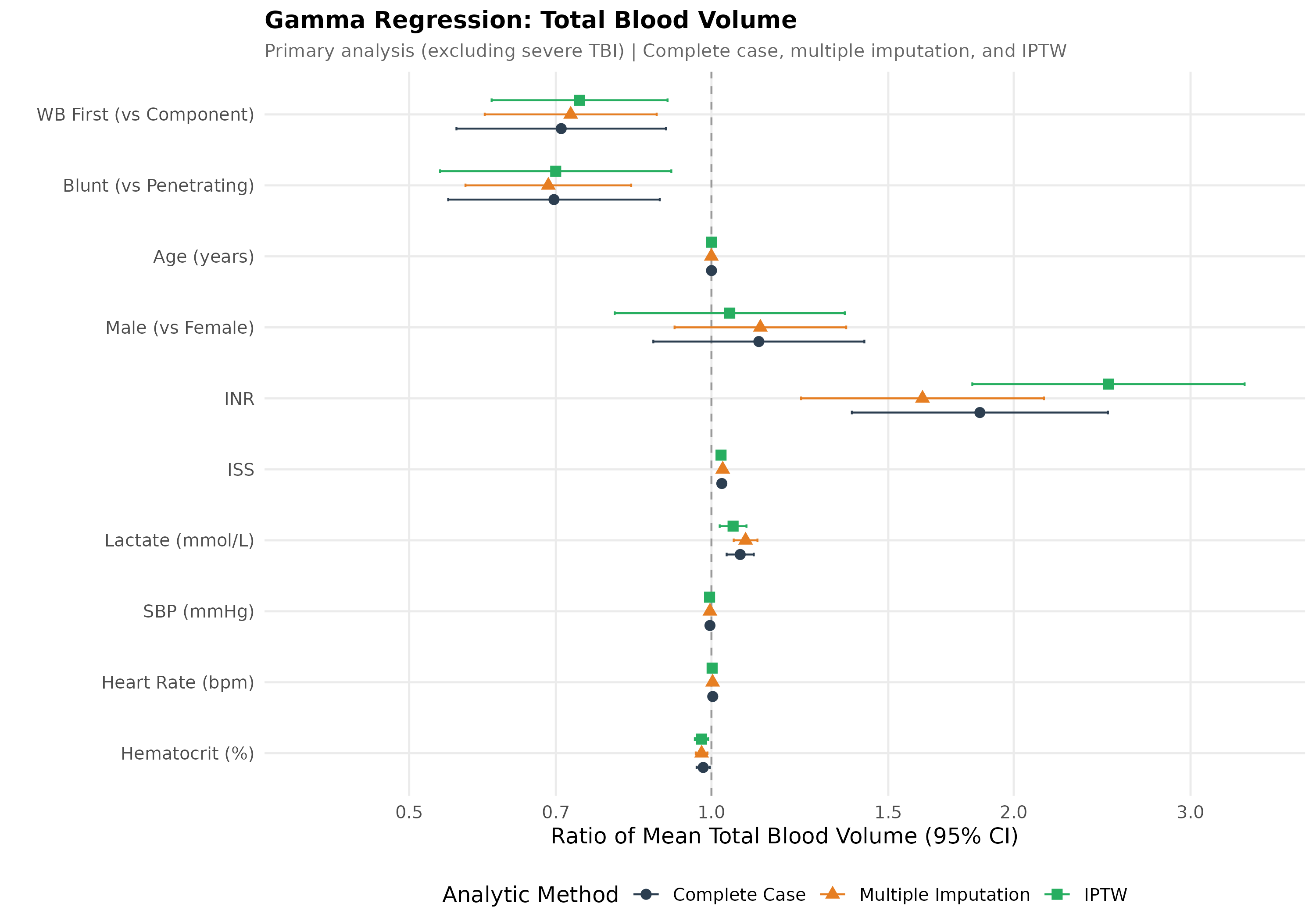


Supplementary Figure 3. Combined forest plot of gamma regression for total blood volume across analytic methods: complete case, multiple imputation, and IPTW (primary analysis, excluding severe TBI).

### Supplementary Figure 4


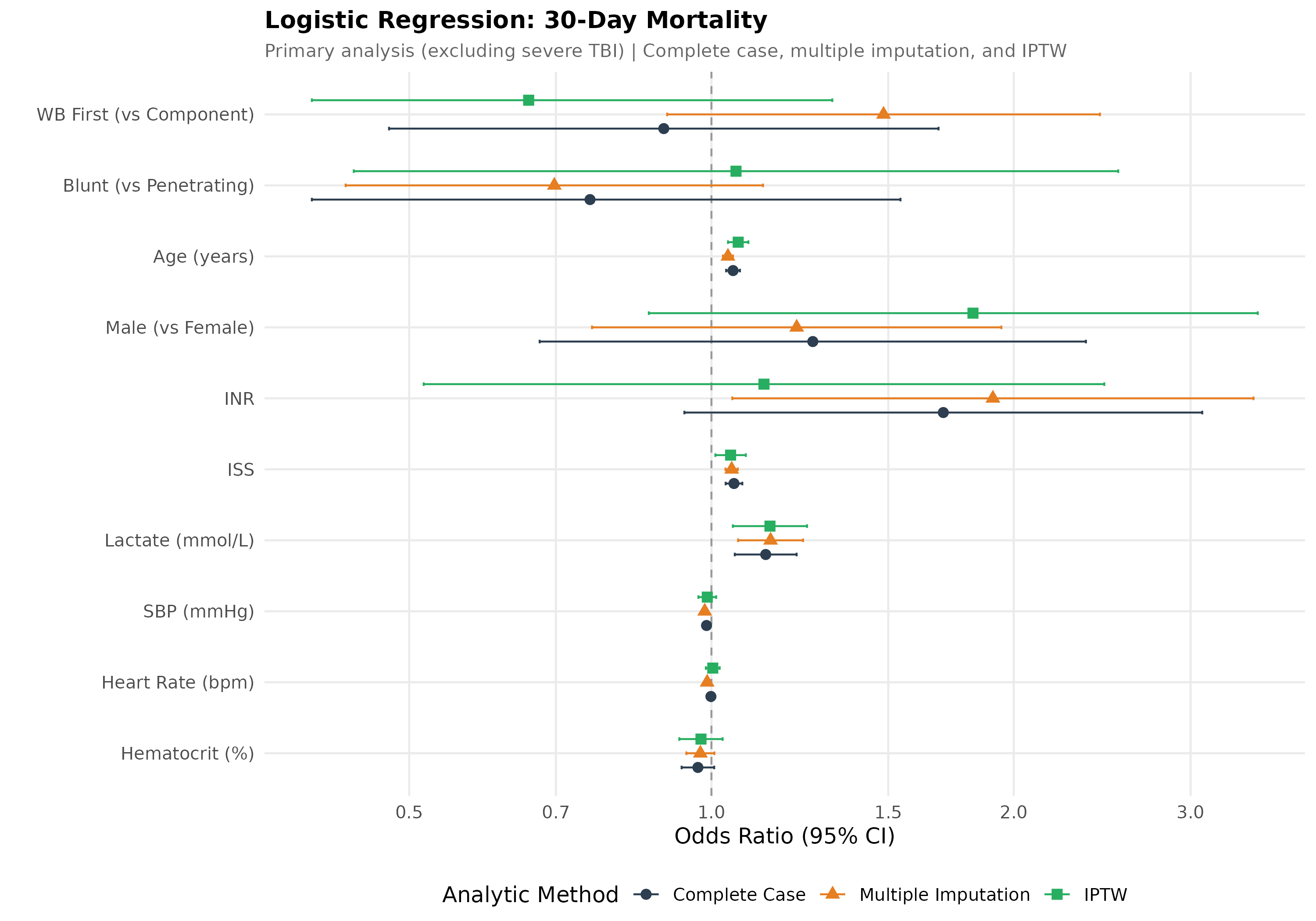


Supplementary Figure 4. Combined forest plot of logistic regression for 30-day mortality across analytic methods: complete case, multiple imputation, and IPTW (primary analysis, excluding severe TBI).

### Supplementary Figure 5


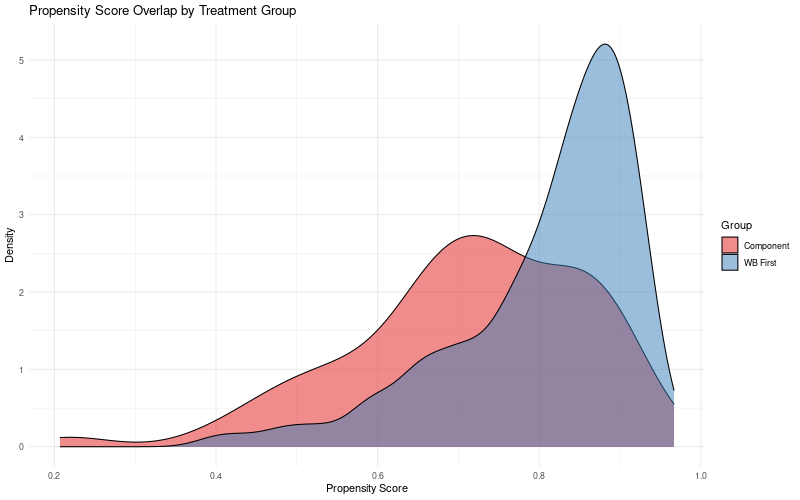


Supplementary Figure 5. Propensity score overlap between WB first and components first groups.

### Supplementary Figure 6


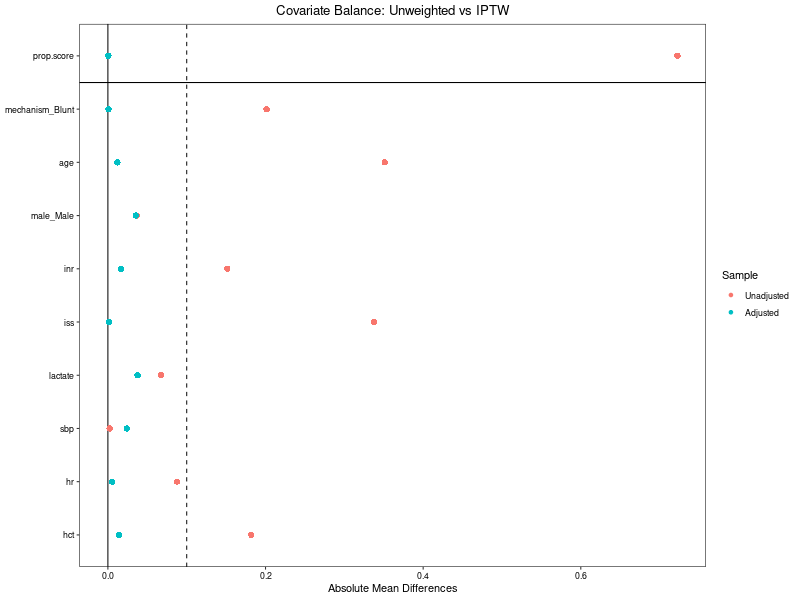


Supplementary Figure 6. Covariate balance before and after IPTW weighting. All standardized mean differences <0.10 after weighting.

References:

1. Li, F., Morgan, K. L., & Zaslavsky, A. M. (2018). Balancing Covariates via Propensity Score Weighting. Journal of the American Statistical Association, 113(521), 390–400. https://doi.org/10.1080/01621459.2016.1260466
